# Electricity production and the analysis of the anode microbial community in a constructed wetland-microbial fuel cell†

**DOI:** 10.1039/c8ra10130b

**Published:** 2019-07-10

**Authors:** Guozhen Wang, Yating Guo, Jiaying Cai, Hongyu Wen, Zhen Mao, Hao Zhang, Xin Wang, Lei Ma, Mengqin Zhu

**Affiliations:** School of Life Science, Jiangsu Normal University Xuzhou 221116 China wenhy@jsnu.edu.cn; School of Environment Science and Spatial Informatics, China University of Mining and Technology Xuzhou 221116 China

## Abstract

The objective of this study is to assess bioelectricity generation, pollutant removal (COD, ammonium, nitrate) and the bacterial communities on anodes in constructed wetlands coupled with microbial fuel cells (CW-MFCs), through feeding the systems with three different types of synthetic wastewater (system 1: normal wastewater; system 2: ammonium-free wastewater; system 3: nitrate-free wastewater). Three CW-MFCs were operated with different wastewater concentrations and hydraulic retention times (HRTs) over a long time period (6 months). The results indicate that the maximum open circuit voltage (775.63 mV) and maximum power density (0.628 W m^−3^) were observed in system 3 (period 3), and that bioenergy production was inhibited in system 2, when feeding with ammonium-free wastewater continuously. COD removal rates in the three systems were similar during each period and ranged from 82.2 ± 6.8% to 98.3 ± 2.2%. Ammonium removal occurred at the air cathode of the CW-MFCs through nitrification, and a higher level of ammonium removal was found in system 1 (period 3) compared with the others. Meanwhile, denitrification occurred at the anaerobic anode of the CW-MFCs, and a large amount of nitrate was removed effectively. The highest nitrate removal rate was 98.8 ± 0.5% in system 2 (period 3). Additionally, four genera related to electricity generation were detected at the anode: *Geothrix*; *Desulfovibrio*; *Desulfobulbus*; and *Geobacter*. The relative abundances of *Desulfovibrio*, *Desulfobulbus* and *Geothrix* gradually increased during the three periods in system 3, which might be beneficial for bioelectricity generation. Further investigations are needed to optimize the CW-MFC performance and explain the mechanism behind the pollutant degradation and electron motion in the CW-MFCs.

## Abstract 2

The objective of this study is to assess bioelectricity generation, pollutant removal and the bacterial communities on anodes in constructed wetlands coupled with microbial fuel cells, through feeding the systems with three different types of synthetic wastewater.

## Graphical abstract

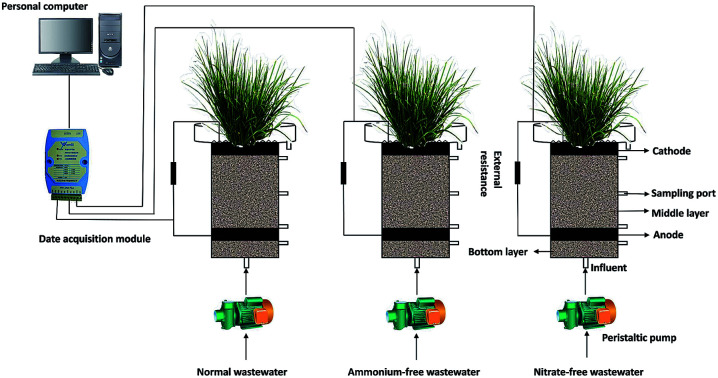

## 1. Introduction

The use of systems involving constructed wetlands coupled with microbial fuel cells (CW-MFCs) is a novel development in the field of environmentally friendly wastewater treatment equipment, contributing to bioelectricity generation and the biodegradation of pollutants.^1–3^ Recently, CWs have been used to treat livestock wastewater containing high levels of ammonium and nitrate.^4^ However, given the limited oxygen transfer rates of traditional CWs and the high residual organic matter and ammonium content in these types of wastewater, treatment efficiency using traditional wetlands is often quite low and requires a large land area.^5^ CW-MFCs are a new technology that couples CWs and MFCs. The surface of a vertical flow CW is an aerobic region and the underlying substrate is an anaerobic region, similar to the cathode and anode of a MFC.^3^ The performance of a MFC can effectively be improved by using a large number of denitrifying microorganisms and electrogenic microorganisms in the CW.^6^ CW-MFCs have the advantages of low cost, easy operation and recyclability, and have been thoroughly studied and widely used in the secondary treatment of domestic sewage, landfill leachate and industrial wastewater.^7^ The structure of a CW-MFC mainly consists of four parts: an anaerobic anode chamber, an intermediate filter layer, an air cathode chamber and macrophytes. The chemical transformations (CH_2_O + *n*CO_2_ → *n*CO_2_ + 4*n*e^−^ + 4*n*H^+^) that take place in the anaerobic anode chamber to degrade pollutants and generate electrons involve a multitude of anaerobic microorganisms and facultative anaerobic microorganisms. The intermediate filter layer mainly functions as a medium for protons to move in and to facilitate the simple filtration of sewage. The reaction at the air cathode is 4*n*e^−^ + *n*/2O_2_ + 4*n*H^+^ → 2*n*H_2_O, which completes the whole electrochemical cycle.^8^ Macrophytes can absorb small amounts of soluble pollutants and provide oxygen for the air cathode.^9^ Furthermore, plant photosynthesis is the main way of promoting bioelectricity generation.

At present, environmental pollution and energy shortages have become serious worldwide problems. The main pollutants are made up of sulfides, organic phosphorus, nitrates, nitrites and ammonium.^10^ For nitrogenous pollutant removal, previous studies have indicated that nitrate/nitrite can be used as an electron acceptor and be removed at the anoxic-cathode chamber of a MFC.^11^ Although nitrate can also be removed at the anode, only weak electricity generation can occur in the presence of nitrate at the anode chamber of a MFC. As a competing electron acceptor, nitrate offers advantages over the anode during the anode reaction, hindering electricity generation.^12^ Srinivasan *et al.* investigated the impact of nitrate at different C/N ratios in mixed-culture chemostats of MFCs. *Geobacter* can simultaneously degrade nitrate and organic pollutants at the anode, producing carbon dioxide and nitrogen and limiting electron donors at lower C/N ratios.^13^ Previous studies have found that the CE is affected by a high C/N ratio, while the maximum voltage output is not significantly affected. MFC bioelectricity generation and nitrate removal functionality are affected by the biofilm thickness of EAB. Sun *et al.* found that power generation and nitrate removal in a MFC first increase and then decrease along with an increase in biofilm thickness.^14^ The removal of nitrates and ammonium under specific conditions has been extensively studied using MFCs.^15–17^ However, whether bioelectricity generation performance is affected by nitrate as a competing electron acceptor in the anode reaction of a CW-MFC has seldom been investigated. Additionally, ammonium should first be nitrified before being removed as an electron acceptor through denitrification in the anoxic-cathode chamber.

Functional microorganisms play an important role in CW-MFC bioelectricity generation and the biodegradation of pollutants. Meanwhile, the bacterial community composition is associated with the removal of pollutants, such as COD, ammonium and nitrate, and electricity generation, and the effects of the bacterial community composition on CW-MFC performance are yet to be investigated.^18^ Since the major oxidation reaction in a CW-MFC occurs at the anaerobic anode, it is particularly important to investigate the microbial community structure of the anode. However, operating conditions such as temperature, HRT, substrate concentration and pH are considered to be important factors that influence the microbial community composition of CW-MFCs. The same utilizable substrate at different concentrations in a MFC might also lead to the bacterial communities developing different structures.^19^ Therefore, the influences of different types of wastewater on the microbial community compositions in CW-MFCs should be accounted for in the future. To date, previous studies have reported certain electrochemically active bacteria (EAB), including *Desulfuromonas*, *Pseudomonas*, *Desulfobulbus*, *Thermincola*, *Geothrix*, *Shewanella*, *Klebsiella*, *Enterobacter*, *Rhodopseudomonas*, *Citrobacter* and *Geobacter*,^20,21^ while most EAB are also nitrobacteria and denitrifying bacteria, such as *Shewanella* and *Geobacter*.^22^ During the denitrification process, the electrons produced by EAB can be used to convert nitrate into nitrogen gas, which can contribute to nitrate removal in CW-MFCs.^23^

In this study, bioelectricity generation, COD removal, ammonium removal, nitrate removal and the bacterial community on the anode were assessed in CW-MFC systems using three different types of synthetic wastewater (system 1: normal wastewater; system 2: ammonium-free wastewater; and system 3: nitrate-free wastewater). The three CW-MFCs operated under continuous influent mode for a long time using different wastewater concentrations and HRTs. We examined and compared the bioelectricity generation performances, wastewater treatment performances and anodic bacterial communities over three periods. The aim of this study was to evaluate the relationships between bioenergy production, pollutant removal efficiency, and microbial community structure in CW-MFCs using different types of synthetic wastewater. Correlations were found between the anode microbial community structure and bioelectricity generation, as well as between the anode microbial community structure and pollutant removal. The effects of the anode microbial community structure on CW-MFC production performance and pollutant removal were also investigated.

## 2. Materials and methods

### 2.1 CW-MFC construction

Three identical vertical flow constructed wetland microcosms were constructed in this experiment. The CW-MFCs consisted of a single cylindrical polyacrylic plastic chamber (internal diameter: 28 cm; height: 50 cm; and volume: about 30 L) containing four layers (bottom layer, anode layer, middle layer and cathode layer) from bottom to top. The bottom layer (5 cm high) and middle layer (20 cm high) were filled with gravel and the average particle size of the gravel was 0.5–1 mm. The average porosity is around 0.2, which resulted in a net liquid volume of about 6 L for each system. The anode layer (10 cm high) and cathode layer (10 cm high) were made of active carbon granules (ACGs) and stainless-steel mesh (SSM). ACGs and SSM are widely used as CW-MFC electrode materials because of their good electrical conductivity.^24^ The SSM was buried in the ACGs as a compound electrode to strengthen electron transfer. Meanwhile, the cathode electrode was located in an overlying water layer on the surface to use oxygen from the atmosphere for the reduction reaction. Four sampling ports were arranged in the middle of each layer throughout the depth of the CW-MFCs to collect samples at different depths. *Ipomoea aquatica* was transplanted into the cathode layer as a cathode plant to absorb pollutants and provide oxygen for the CW-MFC. The anode was led out using titanium wire (diameter: 1 mm) passing through the middle of the reactor and connected to the cathode *via* copper conductors with a resistance of 1000 Ω.

### 2.2 Inoculation and operation

Three sets of CW-MFCs were seeded with active sludge into the anode, and they were prepared and operated under the same conditions. Active sludge was collected from the China University of Mining and Technology Wastewater Treatment Plant (Xuzhou, China). Prior to the start-up of all the systems, the active sludge was pretreated through mixing with ACGs (ACG/active sludge ratio = 3 : 1) and introduced into the anode layers of the CW-MFCs.^25^ After inoculation, three CW-MFCs were provided with nutrient solution for system stabilization during the first month. The nutrient solution contained a carbon source, 5.0 mM phosphate buffer solution (PBS), and trace element solution (1 mL L^−1^). Glucose (600 mg L^−1^) was used as the carbon source for the anodic microorganisms, and 5.0 mM PBS was composed of NaH_2_PO_4_ (4.97 g L^−1^), Na_2_HPO_4_ (2.75 g L^−1^), KCl (0.13 g L^−1^), NaHCO_3_ (3.13 g L^−1^) and NH_4_Cl (0.31 g L^−1^). The concentrated trace element solution (1 mL L^−1^) consisted of (NH_4_)_2_SO_4_ (5.6 g L^−1^), MgSO_4_·7H_2_O (2 g L^−1^), MnSO_4_·2H_2_O (200 mg L^−1^), H_3_BO_2_ (3 mg L^−1^), CoCl_2_·6H_2_O (2.4 mg L^−1^), CuCl_2_·2H_2_O (1 mg L^−1^), NiCl_2_·6H_2_O (2 mg L^−1^), ZnCl_2_ (5 mg L^−1^), FeCl_3_·6H_2_O (10 mg L^−1^), and Na_2_MoO_4_·2H_2_O (0.4 mg L^−1^).^22^ The nutrient solution was continuously added into the three systems through the bottom inlet of the reactor with a peristaltic pump; it then passed through the bottom layer, anode layer, middle layer, and cathode layer, and finally left the system through an upper outlet. The influent flow rate was controlled *via* a peristaltic pump, maintaining an average HRT of 1 day for all systems.

The three prepared different types of synthetic wastewater were continuously pumped into each system when all systems were stable (a reproducible voltage was observed). The three types of synthetic wastewater were normal wastewater, ammonium-free wastewater and nitrate-free wastewater. The compositions and parameters of the three types of synthetic wastewater are shown in Tables S1–S3† (the wastewater in Table S1† was added during period 1, that in Table S2† was added during period 2 and that in Table S3† was added during period 3). In addition, the pH values of the synthetic wastewater were adjusted through adding phosphate buffer (KH_2_PO_4_ and Na_2_HPO_4_), remaining between 7.01 ± 0.22 and 7.56 ± 0.31. A COD/N ratio of 10 : 1 mitigated the limitation of carbon resources during the denitrification process.^26^ Normal wastewater and ammonium-free wastewater were used in systems 1 and 2, and nitrate-free wastewater was fed into system 3 during period 1 (two months; HRT = 1 d). The operating procedures for period 2 (two months; HRT = 2 d) and period 3 (two months; HRT = 3 d) were similar to that of period 1. In this study, all systems were installed in a plant culture room and the air temperature was controlled at 25 ± 1 °C. To avoid uncertain influences on the operating procedures, all periods were repeated 3 times and error bars were used to represent standard errors from parallel experiments.

### 2.3 DNA extraction and high-throughput sequencing analysis

At the end of each period, biofilm samples around the anode electrodes (±1 cm) of the three reactors were collected in 100 mL sterile bottles. All samples from CW-MFCs were centrifuged at a speed of 200 rpm for 12 min in a sterile bottle (100 mL). The supernatant was removed *via* centrifugation and the pellet of each sample was collected for DNA extraction using an E.Z.N.A. stool DNA kit (Omega Bio-Tek, USA), according to manufacturer protocols, and stored at 80 °C for PCR. Trans Start Fast pfu DNA polymerase and ABI Gene Amp 9700 PCR Amplifier were used for PCR amplification. The V4 and V5 hypervariable regions of 16S rRNA were amplified *via* PCR using the universal bacterial primers 515F (5′-GTGCCAGCMGCCGCGG-3′) and 907R (5′-CCGTCAATTCMTTTRAGTTT-3′). The PCR reaction conditions were as follows: initial denaturation at 95 °C for 5 min, followed by 27 cycles of 95 °C denaturation for 30 s, 55 °C annealing for 30 s, and 72 °C extension for 45 s. Finally, extension at 72 °C was carried out for 10 min and at 10 °C until halted. The V4 and V5 PCR amplicons of all samples were sequenced using the Illumina Miseq platform; the amplification and sequencing services were provided by Personal Biotechnology Co., Ltd. (Shanghai, China). To ensure high-quality sequence data, reads were denoised and checked, then removed if homopolymer runs exceeded 8 base pairs (bp) or the reads were shorter than 150 bp, or sequence reads contained chimera sequences or ambiguous bases. All sequence reads were clustered into operational taxonomic units (OTUs) with the UBLAST classifier on the Usearch platform. OTUs were defined with a similarity threshold of 97%.

### 2.4 Analysis and calculations

The voltages generated in the CW-MFCs were monitored through a data acquisition module (DAM-4586, Art Technology Co., Ltd., China) in terms of the voltage drop across an external resistor (1000 Ω) and recorded using a personal computer every 30 min to check the stability of bioelectricity generation. The performances of the CW-MFCs were judged from the power density and polarization curves. Power density (*P*, W m^−3^) was determined through the basic electrical calculation*P* = *U*^2^/*RV*where *U* is the voltage (V), *R* is the external resistance (Ω) and *V* is the volume of the anode electrode. Polarization tests, performed while varying the external resistance over the range of 5 Ω to 100 000 Ω, were employed in this experiment to study the power densities of the CW-MFCs. The coulombic efficiency (CE, %) represents the fraction of electrons used for electricity generation *versus* the electrons in the consumed organic matter, and CE was calculated through the following formula:CE = *MI*/(*nFQ*_in_ΔCOD)where the *M* is the molar mass of O_2_ (32 g mol^−1^). *I* is the current (A) and *n* is the number of electrons donated per mole of O_2_. *F* is the Faraday constant (96 485 C mol^−1^) and *Q*_in_ is the volumetric influent flow rate of the CW-MFC (L s^−1^). Finally, ΔCOD represents the change in COD between the influent and effluent (g L^−1^). The electrode potential was determined against a saturated Ag/AgCl electrode. COD, nitrate, ammonium and the pH of the influent and effluent were investigated during three periods of steady operation. COD, nitrate and ammonium were determined using an ultraviolet spectrophotometer (HACH, DR/2400) according to standard operating procedures. pH was measured with a pH meter (Fisher AR 15, Thermo). The COD removal efficiency (*η*COD) was calculated according to the formula:*η*COD = (COD_in_ − COD_ef_)/COD_in_ × 100%where COD_in_ represents the initial COD concentration (mg L^−1^) and COD_ef_ represents the effluent COD concentration (mg L^−1^). The nitrate and ammonium removal efficiencies (*η*NO_3_^−^–N and *η*NH_4_^+^–N) were calculated using a similar formula to that for *η*COD. One-way analysis of variance (ANOVA) was performed with the SPSS (v22) package to evaluate COD removal, nitrate removal, ammonium removal and the bioenergy production performance in CW-MFCs; *p* < 0.05 was considered as a significant level.

## 3. Results

### 3.1 Bioelectricity generation

**Fig. 1:**
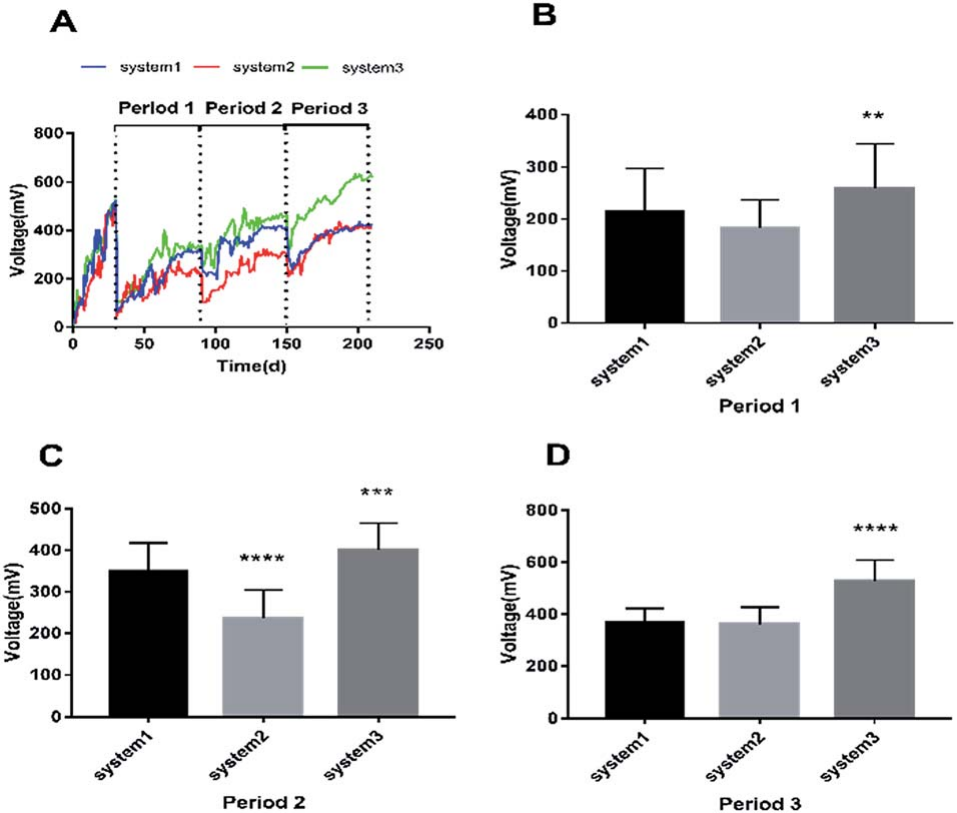
The cell voltage performances of the three systems. (A) Cell voltage variations of the three systems during periods 1, 2 and 3. (B), (C) and (D) Comparisons of the average voltages between the three systems in periods 1, 2 and 3. The error bars indicate standard deviation. An asterisk (*) denotes significant difference (*p* < 0.05) between system 1 and another system.

In order to evaluate the bioenergy produced by the CW-MFCs using different types of synthetic wastewater, voltage data from three devices were continuously recorded over time during each cycle, as shown in Fig. 1A. It is clearly shown that the voltage demonstrated a comparatively high increasing trend in the three CW-MFCs, and the stable voltages of system 1, system 2 and system 3 during acclimation (the first month) were 522 ± 21 mV, 506 ± 18 mV, 518 ± 41 mV, respectively. This phenomenon indicates that CW-MFCs have excellent electricity production abilities when nutrient solutions are utilized as the influent. During period 1, sudden drops in the voltages of the three systems were observed when synthetic wastewater was added into the CW-MFCs, evidently demonstrating that the COD concentration is limited in the feed solution and that electricity production will be weak. This phenomenon also implies that the COD concentration plays a significant role in indicating electricity production potential. The approximate average voltages of systems 1, 2 and 3 were 214.75 mV, 183.53 mV and 259.07 mV, respectively. The average voltage of system 3 was significantly higher than that of system 1 (*p* < 0.0073), while there was no significant difference between the voltages of system 1 and system 2 (Fig. 1B). During period 2, the approximate average voltages of systems 1, 2 and 3 were 349.44 mV, 236.82 mV, and 410.38 mV, respectively. The average voltage of system 3 was significantly higher than that of system 1 (*p* < 0.0002), and the average voltage of system 2 was significantly lower than that of system 1 (*p* < 0.0001) (Fig. 1C). During period 3, the average voltage of system 3 was higher than that of system 1 (*p* < 0.0001), and the average voltage of system 2 showed no significant difference with that of system 1 (Fig. 1D).

**Fig. 2:**
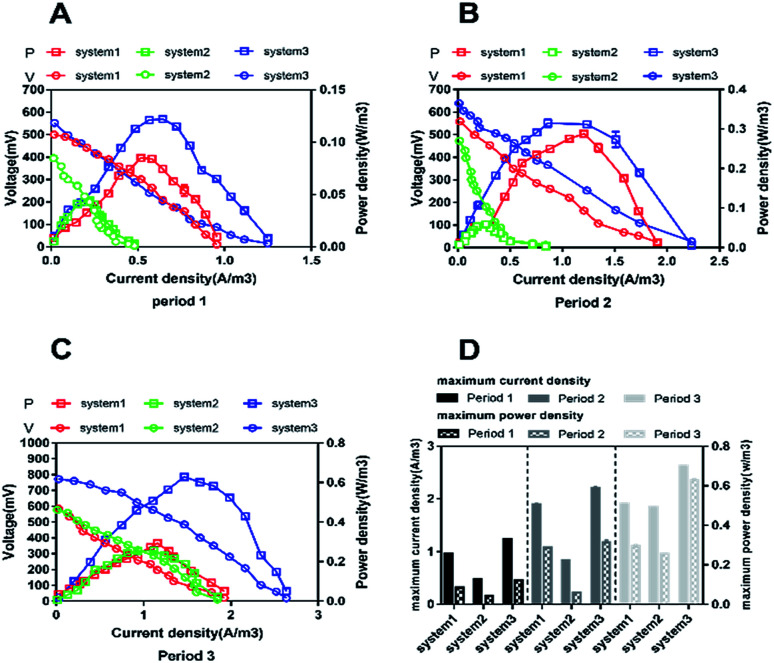
The cell polarization curves and power densities of the three systems. (A), (B) and (C) Polarization curves from periods 1, 2, and 3, respectively. (D) A comparison of the maximum current density and maximum power density values between the three systems during periods 1, 2 and 3.

The construction of polarization curves was the main objective of this study, in order to explore the effects of different types of synthetic wastewater on the electrical performances of the CW-MFC systems. Polarization tests were conducted during the plateau phase for each CW-MFC during every period. During period 1 (Fig. 2A), the highest open circuit voltage of 522.03 mV and the highest power density of 0.121 W m^−3^ were obtained when the current density and the external resistance were 0.42 A m^−3^ and 400 Ω in system 3. Meanwhile, the highest open circuit voltage and the highest power density in system 1 were 500.46 mV and 0.848 W m^−3^, respectively. With the addition of ammonium-free wastewater to system 2, obviously low open circuit voltage and power density values were recorded (394.93 mV and 0.043 W m^−3^, respectively). Compared with period 1, the open circuit voltages during period 2 increased by 10.3% (system 1), 17.5% (system 2), and 16.3% (system 3). The highest power density (0.312 W m^−3^) was obtained in system 3 (Fig. 2B). Similar trends were also observed during period 3 (Fig. 2C). The highest open circuit voltage and the highest power density values for system 1, system 2 and system 3 were 584.76 mV and 0.299 W m^−3^, 578.51 mV and 0.258 W m^−3^, and 775.63 mV and 0.628 W m^−3^, respectively. Fig. 2D clearly shows that different types of synthetic wastewater influence the bioelectricity generation performances of the CW-MFCs. It is evident that system 3 shows great performance, with maximum power density and maximum current density, when nitrate-free wastewater was fed into the CW-MFC during each period. However, compared with system 1 and system 3, the maximum power density and peak current density obtained in system 2 were very low.

CE is an important parameter for evaluating the efficiency of the conversion of organic matter into electrons in CW-MFCs. In our study, low CE was observed in all systems (Table S4†). During period 1 (COD: 275–301 mg L^−1^, HRT: 1 d), the CE range obtained in system 1 was 0.5–0.62%, in system 2 it was 0.3–0.42% and in system 3 it was 0.52–0.85%. During period 2 (COD: 521–588 mg L^−1^, HRT: 2 d), the CE values obtained were 0.46–0.59%, 0.38–0.47% and 0.5–0.47% for system 1, system 2 and system 3, respectively. During period 3 (COD: 822–851 mg L^−1^, HRT: 3 d), all systems presented low CE values in the range of 0.16–0.41%. System 3 showed the highest CE (0.3–0.41%) compared with the others, while a higher COD led to a lower CE to some extent.

### 3.2 The degradation of synthetic wastewater

#### 3.2.1 COD monitoring and COD removal

Each system was continuously fed with different types of synthetic wastewater, which contained designated glucose concentrations, ammonium concentrations and nitrate concentrations, during the three periods. The effluent quality and related parameters from each system were examined when each system gradually became stable (during the middle and late stages of each period), and each sample was restudied 3 times, in order to ensure the accuracy of the data. Correspondingly, the average COD concentrations and ammonium and nitrate concentrations in the influent and effluent, as well as the average COD removal performance of each system over the entire experimental period are shown in Table 1. The COD removal performance is an important index used to evaluate the efficiency of wastewater treatment in CW-MFCs. During period 1, the COD concentrations of the influent and effluent of all systems were analyzed using one-way ANOVA statistical analysis, and the results indicate significant statistical differences between the influent and effluent COD concentrations for all systems (*p* < 0.05). However, no statistical differences between COD removal were found among the three systems (*p* > 0.05) (system 1: 93.2 ± 1.2%; system 2: 91.1 ± 4.8%; system 3: 92.5 ± 3.6%). This phenomenon indicates that although different types of synthetic wastewater could change the microbial community structures of the anodes in CW-MFCs, there is still an abundance of EAB and heterotrophic microorganisms that can utilize organic matter from synthetic wastewater as fuel for metabolic purposes and electricity generation. In addition, the naturally superior adsorption ability of active carbon granules is a critical feature for COD removal. This is in agreement with various studies found in the literature that indicate that the presence of active carbon has a significant impact on the reduction of organic matter and contributes to the biodegradation of organic matter by microorganisms in CW-MFCs. During period 2, the COD removal values of the three systems were slightly higher than during period 1. However, no statistical differences were found between the systems (*p* > 0.05). During period 3, the COD removal values of system 1, system 2 and system 3 were 84.7 ± 2.8%, 88.4 ± 4.8% and 82.2 ± 6.8%, respectively, which are slightly lower than during period 1 and period 2.

**Table 1:** The water quality parameters between the influent and effluent with different types of synthetic wastewater during three periods^a^

Period	CW-MFC	COD concentration (mg L^−1^)				NH_4_^+^ concentration (mg L^−1^)				NO_3_^−^ concentration (mg L^−1^)				pH (−log[H^+^])	Temperature (°C)
		Influent	Anode	Cathode	Removal (%)	Influent	Anode	Cathode	Removal (%)	Influent	Anode	Cathode	Removal (%)		
Period 1	System 1	275.66 ± 34	72.5 ± 12.9	18.7 ± 11.5	93.2 ± 1.2*	26.7 ± 8.7	21.5 ± 3.2	3.1 ± 1.2	88 ± 2.6*	21.6 ± 3.6	4.6 ± 1.2	1.1 ± 0.3	94.9 ± 3.1*	7.2 ± 0.3	23 ± 1.4
	System 2	301.74 ± 53	112.3 ± 28	26.8 ± 18.7	91.1 ± 4.8*	—	—	1.1 ± 0.3	—	31.6 ± 1.4	1.6 ± 0.2	0.7 ± 0.1	97.7 ± 1.6*	6.74 ± 0.2	22 ± 2.5
	System 3	298.53 ± 49.2	88.5 ± 19.7	22.3 ± 14.5	92.5 ± 3.6*	24.8 ± 3.2	19.7 ± 2.8	2.2 ± 1.4	91.1 ± 3.6*	—	—	0.6 ± 0.3	—	6.82 ± 0.4	21 ± 1.2
Period 2	System 1	551.2 ± 41.5	144 ± 15.4	11.2 ± 3.8	98 ± 1.8*	48.6 ± 2.3	38.7 ± 4.4	4.2 ± 2.3	91.3 ± 4.7*	42.6 ± 2.2	3.8 ± 2.2	5.8 ± 2.1	86.4 ± 2.1*	7.4 ± 0.5	22 ± 2.2
	System 2	588.4 ± 55	89.6 ± 44.2	9.7 ± 2.2	98.3 ± 2.2*	—	—	0.7 ± 1.2	—	54.4 ± 1.4	5.1 ± 1.6	1.4 ± 3.3	97.4 ± 3.3*	7.22 ± 0.6	22 ± 2.1
	System 3	521.6 ± 34	124.6 ± 22.9	18.6 ± 8.7	96.4 ± 2.1*	42.6 ± 4.5	28.4 ± 3.2	5.5 ± 6.1	87 ± 2.2*	—	—	1.1 ± 0.2	—	7.7 ± 0.2	23 ± 2.1
Period 3	System 1	822.4 ± 32.1	233 ± 41.5	125.4 ± 15.6	84.7 ± 2.8*	87.6 ± 1.6	62.7 ± 5.1	5.1 ± 2.4	94.1 ± 3.8*	87.8 ± 3.8	4.8 ± 5.5	46.5 ± 4.8	47 ± 5.5*	7.54 ± 0.3	23 ± 2.3
	System 2	851 ± 38	198 ± 36.5	98.7 ± 27.6	88.4 ± 4.8*	—	—	1.1 ± 0.6	—	94.6 ± 4.2	5.8 ± 3.5	1.1 ± 0.2	98.8 ± 0.5*	7.08 ± 0.1	23 ± 3.8
	System 3	844.3 ± 27.9	211 ± 17.4	149.6 ± 21.6	82.2 ± 6.8*	86.5 ± 3.8	71.8 ± 4.4	11.2 ± 1.1	87 ± 3.1*	—	—	0.8 ± 4.4	—	7.21 ± 0.2	24 ± 1.1

a Values are mean results for COD, ammonium and nitrate concentration, pH and temperature data; mean ± standard error. An asterisk (*) denotes significant difference (*p* < 0.05) between the influent and cathode effluent. (—) represents no ammonium or nitrate data.

#### 3.2.2 Ammonium (NH_4_^+^) monitoring and ammonium removal

Organic nitrogen can be effectively removed from sewage in CW-MFCs through a pathway of ammonification (the conversion of organic nitrogen to ammonium), using nitrification (NH_4_^+^ + 2O_2_ → NO_3_^−^ + 2H^+^ + 2H_2_O) and denitrification (NO_3_^−^ + 5e^−^ + 6H^+^ → 0.5N_2_ + 3H_2_0 or NO_3_^−^ + 2e^−^ + 2H^+^ → NO_2_^−^ + H_2_O) processes that incessantly occur at the anode and cathode of a CW-MFC.^27^ Therefore, nitrifying bacteria and denitrifying bacteria play crucial roles in the nitrification and denitrification processes, contributing to nitrogen removal from wetland environments. Additionally, the nitrification process does not remove any nitrogen, it only transforms ammonia into nitrate.^28^ The ammonium concentrations and ammonium removal efficiencies in the CW-MFC systems using different synthetic wastewater types are presented in Table 1. During period 1, the average ammonium removal efficiencies of the CW-MFCs were 88 ± 2.6% (system 1) and 91.1 ± 3.6% (system 3). This result indicates that obviously high ammonium removal efficiencies were achieved in the CW-MFCs. Meanwhile, the ammonium concentrations decreased from 26.7 ± 8.7 mg L^−1^ (influent) to 21.5 ± 3.2 mg L^−1^ (anode) to 3.1 ± 1.2 mg L^−1^ (cathode) and from 24.8 ± 3.2 mg L^−1^ (influent) to 19.7 ± 2.8 mg L^−1^ (anode) to 2.2 ± 1.4 mg L^−1^ (cathode) in system 1 and system 3, respectively. This phenomenon indicates that the main region where ammonium removal takes place is in the cathode of the CW-MFC during this process. Similar ammonium removal performances were observed during period 2 and period 3. The average ammonium removal efficiencies were 91.3 ± 4.7% (system 1, period 2), 87 ± 2.2% (system 3, period 2), 94.1 ± 3.8% (system 1, period 3) and 87 ± 3.1% (system 3, period 3). It can be seen that ammonium removal in the CW-MFCs also varied according to different HRTs and synthetic wastewater concentrations, despite these differences not being significant (*p* > 0.05).

#### 3.2.3 Nitrate (NO_3_^−^) monitoring and nitrate removal

Systems 1 and 2 removed nitrate with similarly high efficiencies (94.9 ± 3.1% and 97.7 ± 1.6%) during period 1. The concentrations of nitrate decreased from 21.6 ± 3.6 mg L^−1^ (influent) to 4.6 ± 1.2 mg L^−1^ (anode) to 1.1 ± 0.3 mg L^−1^ (cathode), and from 31.6 ± 1.4 mg L^−1^ (influent) to 1.6 ± 0.2 mg L^−1^ (anode) to 0.7 ± 0.1 mg L^−1^ (cathode) in system 1 and system 2, respectively. These results indicate that higher nitrate removal efficiencies can be achieved using CW-MFCs through the use of an activated carbon electrode, and that the nitrate in the influent can be mostly removed through the anode in a CW-MFC. A similar phenomenon has been reported in other studies during recent years.^26^ During periods 2 and 3, the nitrate concentrations in the effluents of the CW-MFCs gradually declined between the inlet and anode. Nevertheless, the nitrate concentrations began to gradually increase when synthetic wastewater flowed through the cathode of system 1. This phenomenon may indicate that the diffusion of oxygen and oxygen released from plant roots to the cathode inhibit microbial denitrification and that the large amounts of ammonium in normal wastewater could promote nitrification, leading to nitrate concentration increases at the cathode.

### 3.3 Bacterial community structures at the anode

#### 3.3.1 The richness and evenness of bacterial community structures in anode biofilms

The bacterial communities from biofilm samples from each anode of the CW-MFCs were collected and then pyrosequencing-based analysis was performed on them. 30099-57724 high-qualified 16S rRNA effective sequence tags with a dominant length range of 351–400 bp were obtained from each sample. The richness and evenness of the microbial communities in the three CW-MFCs using different types of synthetic wastewater are shown in Table 2. The number of operational taxonomic units (OTUs) on the inoculum from active sludge was 887, which was significantly different when compared with other samples from different periods (*p* < 0.01). Totals of 1721, 1817 and 1750 OTUs were identified at 97% similarity in the anode biofilms of the CW-MFCs during period 1, when supplemented with normal wastewater, ammonium-free wastewater and nitrate-free wastewater, respectively. During period 2, OTU values of 1760 (system 1), 1582 (system 2) and 1951 (system 3) were obtained from the anode biofilms of each CW-MFC. The OTU numbers varied from 1728 (system 3) to 1840 (system 2) during period 3. The Simpson and Shannon indices were used evaluate the abundance and uniformity of the microbial communities. The anode biofilm bacterial communities with normal wastewater had relatively higher diversity (Shannon index of 6.01; Simpson index of 0.006) than those of the anode biofilms using other synthetic wastewater types (Shannon indices of 5.86–5.88; Simpson indices of 0.0079–0.0083) during period 1. The diversity of the bacterial community of system 3 was similar to that of system 1 during period 2. During period 3, the Simpson diversity index values increased in the following order: system 2 < system 3 < system 1. In addition, the Shannon diversity estimator values were in a close range between 5.84 (system 2) and 5.86 (system 1), similar to the anodic biodiversity of CW-MFCs previously reported.^29^ The highest abundance and uniformity of microbial communities were found in system 2 for all experiments. This result indicates that normal wastewater might contribute to high microbial diversity. In addition, system 3 and system 1 had high Shannon–Weaver indices during period 2, which coincided with the highest maximal power output being generated. This result may be attributed to electricity generation by microorganisms. Furthermore, the ACE or Chao1 estimator was used to evaluate the community richness of the bacterial communities.^30^ In general, the Chao1 estimator values of system 1 were apparently higher than those of system 2 and 3 during all periods, indicating that the richness of bacterial structures might be affected by ammonium and nitrate during the process of bioelectricity generation in CW-MFCs. During period 2, the Chao1 estimator ranged from 2037 (system 2) to 2339 (system 3), while the richness of the bacterial communities of the three devices decreased in the following order: system 3 > system 1 > system 2. A similar trend was obtained for the power densities of the CW-MFCs.

**Table 2:** The richness and evenness of the microbial community compositions on the anodes of the three systems^a^

Period	System	OTU	Shannon	Simpson	Chao1	Coverage
	Inoculum	877	4.23	0.0355	1101	0.995799
Period 1	System 1	1821	6.01	0.006	2228	0.991429
	System 2	1817	5.86	0.0083	2235	0.989278
	System 3	1750	5.88	0.0079	2100	0.986283
Period 2	System 1	1760	5.89	0.008	2188	0.985915
	System 2	1582	5.78	0.0081	2037	0.985348
	System 3	1951	5.88	0.0073	2339	0.992274
Period 3	System 1	1833	5.86	0.009	2338	0.986109
	System 2	1840	5.84	0.0082	2224	0.988577
	System 3	1728	5.86	0.0083	2140	0.984119

a A higher Chao1 diversity index value represent more richness in the bacterial community. Meanwhile, high Shannon and Simpson index values represent more richness and evenness in the bacterial community.

**Fig. 3:**
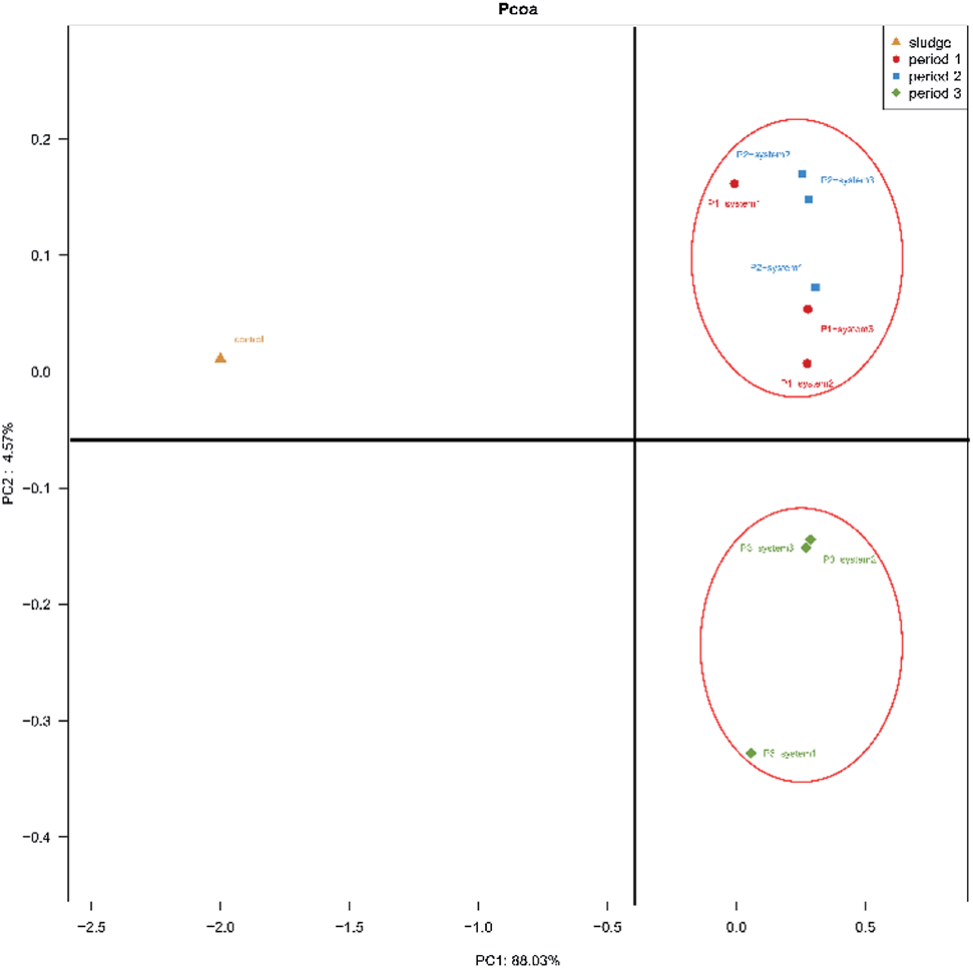
Differences in microbial community structure distributions on the surfaces of anode electrode samples between the three systems during all periods, as indicated by weighted fast UniFrac PCoA based on phylogenetic lineages.

The differences in microbial community compositions between the three systems were evaluated using weighted fast UniFrac PCoA analysis based on their phylogenetic lineages (Fig. 3). PC1 and PC2 accounted for 88.03% and 4.57% of the distinguished variabilities in the microbial community structures. As seen in Fig. 3, six samples obtained during period 1 and period 2 were clustered together and were separated from three samples obtained during period 3 and the inoculum. The results indicate that the HRT and synthetic wastewater concentration affected the microbial communities of the anode biofilms in the MFCs. Simultaneously, this phenomenon also indicated that the microbial communities of all the CW-MFCs were similar during periods 1 and 2. The microbial communities that developed in the inoculum were distinctly different from those of other samples obtained from the CW-MFCs. This result indicates that the microbial communities of the active sludge changed when it was inoculated into the CW-MFCs in these experiments.

#### 3.3.2 Bacterial community compositions of anode biofilms in CW-MFCs

**Fig. 4:**
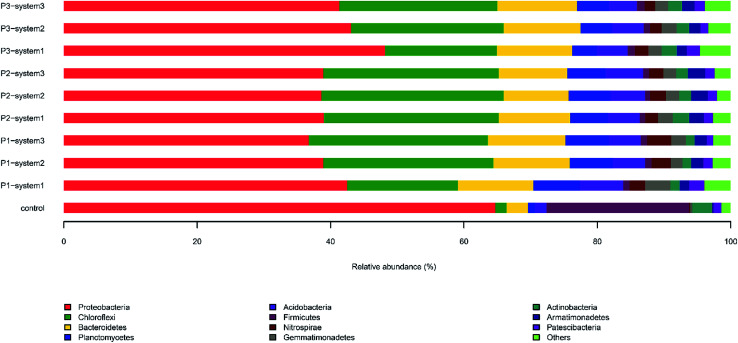
The relative abundances of 16S rDNA sequences in anode biofilm samples from the CW-MFCs supplemented with different types of synthetic wastewater during three periods at the phylum level.

The microbial community compositions (at the phylum level) of the anodes in response to different types of synthetic wastewater are shown in Fig. 4. It is clear that the bacterial community composition of the inoculum, in which the major dominant phyla included Proteobacteria, Firmicutes and Actinobacteria, was distinctly different from those of the other samples. The levels of Chloroflexi in the CW-MFCs clearly outweigh that of the newly inoculated sludge (control). The Chloroflexi phylum shows the ability to acquire energy sources through halogenating organics (such as polychlorinated biphenyls and chlorinated ethenes) under aerobic and anaerobic conditions.^31^ Proteobacteria, Chloroflexi, Bacteroidetes and Planctomycetes were the most predominant phyla in the anodes of the CW-MFCs. The relative abundance of Proteobacteria in the anode biofilm of system 1 (42.3%) was higher than that of system 2 (39.4%) and system 3 (36.6%). Previous studies have demonstrated that Proteobacteria are defined as EAB in MFC reactors.^20^ Similar exoelectrogenic bacteria species have been reported in previous studies.^32^ Compared with period 1, the relative abundances of Proteobacteria in system 1 and system 2 decreased during period 2. Nevertheless, the relative abundance of Proteobacteria increased in system 3. During period 3, the relative abundances of Proteobacteria in the anode biofilms gradually increased in all three CW-MFCs (system 1: 38.9–48.2%; system 2: 38.5–43.1%; system 3: 39–41.3%). A similar trend was observed for Firmicutes and Actinobacteria, which are also defined as EAB.

The major dominant classes in the anode biofilms during period 1 were, in order, Betaproteobacteria (19.7–23.4%) (Fig. S1†), Gammaproteobacteria (7.5–11.2%), Anaerolineae (10–16.6%) and Deltaproteobacteria (5.5–6.7%). The presence of Betaproteobacteria and Gammaproteobacteria, which contribute to a reduction of nitrate and nitrite, was verified.^33^ The relative abundance of Betaproteobacteria in system 3 (19.8%) was lower than in system 1 (20.4%) and system 2 (23.4%). The relative abundance of Betaproteobacteria in system 3 was the lowest during the three periods of this experiment, indicating that the nitrate concentration could affect the relative abundance of Betaproteobacteria in CW-MFCs. During period 2 and 3, the relative abundances of Betaproteobacteria and Gammaproteobacteria grew steadily with an increase in the HRT and the synthetic sewage concentration, and finally stabilized.

On the family level (Fig. S2†), Rhodocyclaceae and Anaerolineaceae were the most predominant families in the anode biofilm samples from the three systems. The relative abundance of Rhodocyclaceae in system 2 (13.3%) was higher than in system 1 (7.9%) and system 3 (10.5%). It is recognized that this family is capable of nitrogen removal through denitrification, *via* a process in which short-chain fatty acids are usually used as electron donors. The level of the family Anaerolineaceae in the anode biofilm of system 2 (16.6%) was similar to that in the anode biofilm of system 3 (16.5%). This family has been identified as acidogenic fermenting bacteria that possess the capacity to remove organic matter from wastewater.^34^ The relative abundances of Rhodocyclaceae sharply increased and were highest in the three CW-MFCs during periods 2 and 3. Notably, the different types of synthetic wastewater resulted in the formation of apparently diverse microbial communities. For example, the relative abundances of Ruminococcaceae and Anaerolineaceae in system 3 were obviously higher than in system 1 and system 2 samples during the three periods. Anaerolineaceae, as an EAB, is predominant in plant-based sediment microbial fuel cells (PMFCs) and it can transfer electrons to the electrode.^35^

**Fig. 5:**
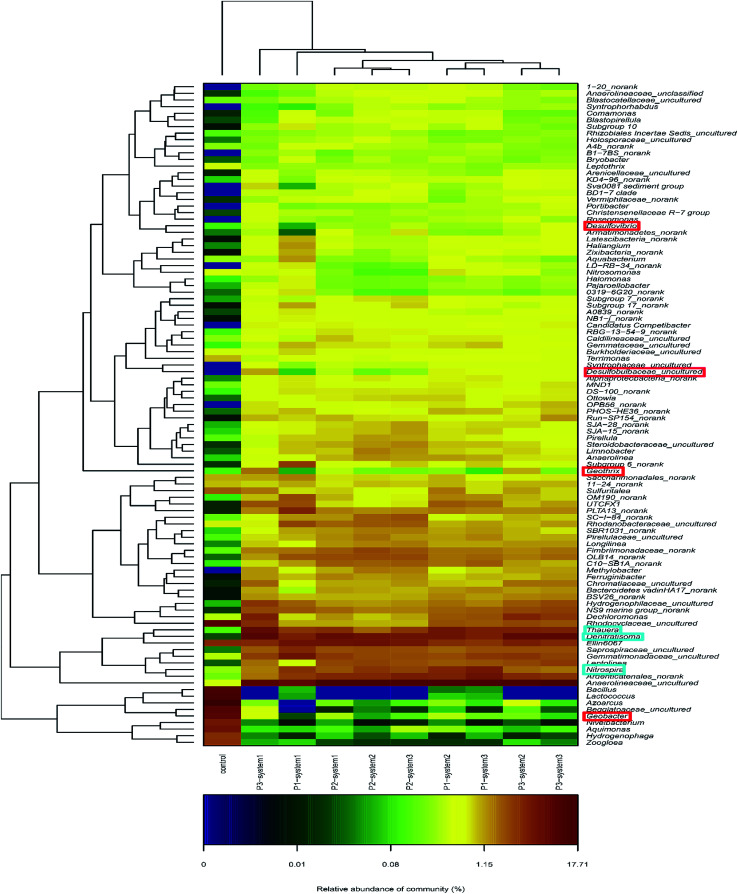
A heat map graph of the hierarchy clusters for the top 100 genera. The color intensity in each panel respects the similarity characteristic between the four samples. Red and blue colors mean the good or poor enrichment, respectively, of a genus.

The microbial community compositions at the genus level are illustrated in Fig. 5. The major dominant genera in the newly inoculated sludge (control) were, in order, *Azoarcus* (14%), *Lactococcus* (10.1%), *Bacillus* (8.8%) and *Geobacter* (6.5%). In contrast, different bacterial community compositions were observed in the anode biofilm samples from the CW-MFCs, in which the major dominant genera included Anaerolineaceae_uncultured (8.5–12.4%), Nitrosomonadaceae_uncultured (3.9–4.4%), *Nitrospira* (2.4–3.6%) and *Thauera* (2.2–3.9%). Seven identified EAB types in the CW-MFCs, *Thermomonas*, *Geothrix*, *Desulfovibrio*, *Desulfobulbus*, *Pseudomonas*, *Geobacter* and *Clostridium*, were discovered and listed.^36,37^ The CW-MFC supplemented with nitrate-free wastewater (system 3) showed the highest relative abundances of *Desulfovibrio*, *Pseudomonas* and *Clostridium*, compared with the CW-MFCs supplemented with normal wastewater (system 1) and ammonium-free wastewater (system 2) during periods 2 and 3. *Geobacter*, defined as an important EAB, was observed in all three CW-MFCs.^20^ During period 1, the relative abundance of *Geobacter* in system 2 was 0.34% higher than in system 1 (0.01%) and system 3 (0.14%). With an increase in the HRT and synthetic wastewater concentration, the relative abundances of *Geobacter* in system 1 (0.29%) and system 3 (0.12%) gradually increased and were higher than in system 2 (0.04%) during period 2. Nevertheless, during period 3, the relative abundance of *Geobacter* sharply dropped in system 3 (0.12–0.06%). The average abundances of the following nitrifying bacteria and denitrifying bacteria were obtained from all three systems: *Nitrospira*; *Thauera*; *Dechloromonas*; *Planctomyces*; *Thermomonas*; *Bacillus*; *Thiobacillus*; *Pseudoxanthomonas*; *Uliginosibacterium*; *Arenimonas*; *Hydrogenophaga*; and *Rhodobacter*. Among these nitrifying bacteria and denitrifying bacteria, *Nitrospira*, *Thauera* and *Dechloromonas* had high relative abundances in all three systems. The relative abundance of *Thauera* gradually increased in system 1 (2.3% → 2.7% → 5.1%) over the three periods. At the same time, we also found that four varieties of microbes that remove organic matter, Cytophagaceae_uncultured, Rhodospirillaceae_uncultured, *Clostridium* and *Propionivibrio* could be identified in the three CW-MFCs. *Propionivibrio* has been reported to be chemoorganotrophic, belonging to the class Betaproteobacteria. The presence of *Clostridium*, a type of glucose, starch or xylose biodegrading bacteria, indicates the enhancement of the organic matter removal capacity around the anodes of the CW-MFCs.

### 3.4 Functional analysis of the microbial communities

PICRURUSt (Phylogenetic Investigation of Communities by Reconstruction of Unobserved States) functional gene prediction analysis is shown in Fig. S3.† Four functional gene groups that were dominant in the three systems were observed over the whole prediction analysis and are as follows: membrane transport (10.7–11.5%); carbohydrate metabolism (9.9–10.3%); amino acid metabolism (10.1–10.3%); and energy metabolism (6.1–6.3%). The functional gene levels for the carbohydrate metabolism of P1-system 1 and P3-system 1 were significantly lower than in the other CW-MFC samples. The carbohydrate metabolism is related to the degradation of carbohydrates. Thus, we speculate that the lowest relative abundance of enzymes leads to a negative effect on sewage treatment in system 1. In terms of energy metabolism, the levels in system 3 were significantly higher than in system 1 and system 2 during all three periods. Energy metabolism is related to energy conversion and metabolism. More biomass energy was converted to electrical energy by EAB in system 3. Therefore, bioelectricity generation in system 3 was significantly higher than in the other systems. During period 3, the levels of functional genes for membrane transport in the three systems decreased in the order: P3-system 3 > P3-system 2 > P3-system 1. Membrane transport is related to the metabolic rates of microorganisms. These phenomena might be able to explain the excellent sewage treatment capacity and power generation capacity of system 3.

## 4. Discussion

### 4.1 Correlation between bioelectricity generation and microbial community composition

The bioelectricity generation mechanism of a CW-MFC is as follows. In the anode, glucose from synthetic wastewater can be used as a resource by EAB to generate electrons (e^−^) and protons (H^+^). Electrons (e^−^) are transferred to the cathode along the external circuit and protons (H^+^) are transferred to the cathode *via* flow. In the cathode, oxygen from the air cathode acts as an electron acceptor and oxidizes with protons (H^+^) and electrons (e^−^) (O_2_ + 4H^+^ + 4e^−^ → 2H_2_0).^37–40^ This study attempted to explore the effects of different types of synthetic wastewater on the bioelectricity generation mechanism of CW-MFCs. In this study, the bioelectricity generation performances of the three CW-MFCs showed significant differences based on the different types of synthetic wastewater used. A negative bioelectricity generation effect was observed in system 2 (0.04–0.258 W m^−3^), which was supplied with ammonium-free wastewater over the entire operation period. This result can lead to the interpretation that nitrate can act as an electron acceptor and compete for the electrons produced by organic matter (OM) oxidation at the anode when synthetic wastewater containing high concentrations of nitrate flows through the anode of the CW-MFC.^38^ The concentration of nitrate in the influent was mostly removed through the anodes of the CW-MFCs. A similar phenomenon has been reported in other studies conducted during the previous year.^26^ Nitrate removal in a CW-MFC is mainly based on a pathway of denitrification (NO_3_^−^ + 5e^−^ + 6H^+^ → 0.5N_2_ + 3H_2_0 or NO_3_^−^ + 2e^−^ + 2H^+^ → NO_2_^−^ + H_2_O). However, the nitrate in the anode layer may consume more electrons during reduction and less electrons in the cathode half-cell reaction, leading the cathode potential to decrease. Therefore, electricity generation in CW-MFCs will be inhibited when using sewage with a high nitrate concentration in the anode. System 3 showed excellent power generation performance during the entire experiment. This is perhaps attributed to: (1) no nitrate or other electron acceptors flowing through the CW-MFC anode region, resulting in a large amount of electrons (e^−^) from EAB-oxidized organic matter enriching the electrode and being passed to the cathode; and (2) the nitrification (NH4^+^ + 2O_2_ → NO_3_^−^ + 2H^+^ + 2H_2_O) of ammonium ions in synthetic wastewater under aerobic conditions in the cathode chamber, where nitrate is formed as an electron acceptor that can promote bioelectricity generation in the CW-MFC.

In this study, glucose and sodium acetate acted as carbon sources, and the bioelectricity generation of all CW-MFCs increased along with an increase in the carbon source concentration during the three periods. The value of bioenergy output increased from 0.08 to 0.28 to 0.3 W m^−3^ from period 1 to period 3 in system 1; from 0.04 to 0.06 to 0.26 W m^−3^ in system 2; and from 0.13 to 0.31 to 0.63 W m^−3^ in system 3. This phenomenon indicates that the higher carbon source concentration available during periods 2 and 3 might facilitate the activity and growth of electrochemically active bacteria. It can be speculated that CW-MFCs may show excellent power generation performance at higher carbon source concentrations.

The low CEs (lower than 2%) reported in previous studies of CW-MFC systems demonstrate an urgent problem that needs to be solved. A negative impact on the CEs was observed when feeding with higher substrate concentrations in all three systems. Similar results have been previous reported.^23^ The main reasons for this are as follows. Firstly, many heterotrophic microorganisms, such as methanogens, are present in the bottom and anode layers of CW-MFCs and they consume large amounts of organic matter, which is converted to methane. During periods 2 and 3, although large COD removal percentages were observed due to biological oxidation, the electrons released were not effectively captured or utilized at the anode. Secondly, in system 1 and system 2, due to high concentrations of nitrates in the anode zone, the electrons used for nitrate removal led to a decrease in the number of electrons available for electricity production,^38^ thus resulting in CE decline.

EAB play an important role in the CW-MFC bioelectricity generation process. Research has shown that the composition and relative abundance of EAB have a strong influence on CW-MFC electricity generation.^18,26^ Seven genera related to electricity generation were identified in the CW-MFCs: *Thermomonas*; *Geothrix*; *Desulfovibrio*; *Desulfobulbus*; *Pseudomonas*; *Geobacter*; and *Clostridium*. Fig. S4† shows the relationship between four important EAB and the bioenergy outputs of the CW-MFCs. After the CW-MFCs had been operating for three periods, the relative abundances of *Geobacter* (0.016% to 0.42%), *Desulfovibrio* (0.03% to 0.34%), *Desulfobulbus* (0.02% to 1%) and *Geothrix* (0.03% to 1.24%) increased with an increase in the substrate concentration and HRT of system 1 (Fig. S4A†). Meanwhile, *Desulfobulbus* and *Geothrix* abundances increased sharply from period 2 to period 3. This phenomenon indicates that a high abundance of EAB can effectively increase the bioelectricity generation abilities of CW-MFCs. As shown in Fig. S4B,† the relative abundances of the genera *Desulfobulbus* and *Geobacter* in system 2 decreased rapidly from 0.005% to 0.0009% and from 0.0034 to 0.0004%, respectively, during period 2, suggesting that *Desulfobulbus* and *Geobacter* enrichment on the anode is inhibited by a lack of ammonium ions. The abundances of these two genera in system 2 decreased significantly from period 1 to 2, which can be interpreted as resulting in the phenomenon of negative bioelectricity generation in system 2. In system 3, the four EAB returned to higher abundances with an increase in the substrate concentration and HRT. As shown in Fig. S4C,† the relative abundances of *Desulfovibrio*, *Desulfobulbus* and *Geothrix* gradually increased during all three periods, which may be beneficial to bioelectricity generation in system 3.

### 4.2 Correlation between the degradation of synthetic wastewater and the microbial communities

Heterotrophic microorganisms can play a significant role in the process of organic matter removal in CW-MFCs. In this study, high COD removal rates were observed in the anode layers of all three systems. Meanwhile, the COD removal performances of all CW-MFCs gradually decreased as the substrate concentrations and HRTs increased. The COD removal performance of a CW-MFC is inhibited when a higher organic matter load is present in the synthetic wastewater.^26^ Illumina Hiseq 16S rRNA gene sequencing showed a large number of heterotrophic microorganisms, including Cytophagaceae, *Uliginosibacterium*, *Propionivibrio*, Bacteroidales and Anaerolinaceae, in the anode layer of the CW-MFCs. The order Cytophagaceae possesses the capability to biodegrade refractory organic compounds, including aromatic compounds, through anaerobic biodegradation, which may promote organic matter removal at the anaerobic anode.^39,40^ The genus *Uliginosibacterium*, which possesses the capability to remove nitrogen and/or organic matter from wastewater,^41^ was also observed in the CW-MFCs. Bacteroidales and Anaerolinaceae also increased in abundance in the CW-MFCs. These families have been identified as acidogenic fermenting bacteria that possess the ability to remove organic matter from wastewater.^34^ Thus, it could be speculated that the COD removal efficiency can be significantly increased with an increased in the abundance of heterotrophic microorganisms in the anode.

According to previous studies, denitrification occurs at the anaerobic anode of CW-MFCs, and nitrate is effectively removed.^27^ Meanwhile, the process of nitrate removal causes a large amount of electrons to be consumed, which leads to the reduction of the anode potential and decreased bioelectricity generation in CW-MFCs.^38,42^ It is very critical that the carbon balance and nitrogen balance are explored for CW-MFCs in relation to wastewater removal. Xu *et al.* discovered multiple carbon metabolism pathways through studying the carbon balance of CW-MFCs.^25^ Therefore, studies of the nitrogen balance in CW-MFCs are urgently needed. Ammonium removal occurs at the air cathode of CW-MFCs through nitrification,^27^ and nitrate from the nitrification process can be used as an electron acceptor to promote electricity generation.^38^ This result explains the presence of nitrate in the air cathode area (Table 1) and the excellent bioelectricity generation performance of system 3. Therefore, nitrifying and denitrifying bacteria play important roles in the removal of nitrogenous wastewater. At genus level, the relative abundances of nitrifying and denitrifying bacteria, including *Planctomyces*, *Nitrospira*, *Bacillus*, *Thauera*, *Dechloromonas*, *Pseudomonas*, and *Thiobacillus*, *Flavobacterium*, were also detected at the anaerobic anode through high-throughput sequencing analysis. Interestingly, we not only found anaerobic denitrifying bacteria in the anaerobic anode region, but also observed some aerobic nitrifying bacteria. This indicates that some small-scale aerobic regions exist in the anaerobic anode region. This result is consistent with the results of a previous study.^18^ Higher proportions of denitrifying bacteria, including *Nitrospira*, *Thauera* and *Dechloromonas*, were observed at the anode (Fig. S5†). It is clear from Fig. S5† that the relative abundances of these three microorganisms fluctuate in different systems and during different periods. *Nitrospira* can play a significant role in nitrifying and denitrifying, and the highest relative abundance of *Nitrospira* was found in system 3 (period 1). Meanwhile, the relative abundances of *Nitrospira* gradually reduced from period 1 to period 3 in all systems. *Thauera* has been identified as a type of autotrophic denitrifying bacteria that is able to biodegrade nitrogen without organic matter. A higher abundance of these bacteria was observed in system 1 (period 1) compared with the other systems. This result demonstrates that system 1 has excellent nitrate removal capacities. In system 2, the relative abundance of *Thauera* decreased rapidly from period 1 to period 2 (0.04% to 0.015%). The average relative abundances of *Dechloromonas*, which has been reported to be a type of autotrophic denitrifying bacteria, phosphate-accumulating microorganism and chlorate-reducing bacteria,^43,44^ were similar in all three systems (period 3). There were a variety of EAB, such as *Geobacter*, *Desulfovibrio* and *Pseudomonas*, at the anaerobic anode. It has been reported that the nitrate removal efficiency can be significantly increased through using the electrons generated in CW-MFC reactors. Additionally, the change in water quality over the entire experimental period in all systems was attributed to adsorption by activated carbon and other substances. The process of the plant uptake of organic matter, nitrate and ammonium through the rhizosphere was observed, but the removal efficiency of nitrate through plant uptake was apparently lower than when carried out by microbes.^45^

## 5. Conclusions

In this study, the optimal operating conditions for CW-MFCs were investigated through evaluating the bioelectricity generation and pollutant removal performances of CW-MFCs under different operating conditions and periods of time. Further correlational research relating to microbial community structures, electricity generation and decontamination under different conditions was undertaken. In terms of bioelectricity generation, the results indicated that the maximum open circuit voltage (775.63 mV) and the maximum power density (0.628 W m^−3^) were observed in system 3 (period 3), and that bioenergy production was inhibited in system 2. It was verified that electricity generation by CW-MFCs would be inhibited using sewage with high nitrate concentrations in the anodes. In terms of pollutant removal, the COD removal rates of the three systems were similar during each period and ranged from 82.2 ± 6.8% to 98.3 ± 2.2%. Ammonium removal occurred at the air cathodes of the CW-MFCs through nitrification, and a higher ammonium removal efficiency was found in system 1 (period 3) compared with the others. Meanwhile, denitrification occurred in the anaerobic anodes of the CW-MFCs, and a large amount of nitrate was removed effectively. The highest nitrate removal rate was 98.8 ± 0.5% in system 2 (period 3). In the anodic microbial communities, four genera related to electricity generation were detected in the CW-MFCs: *Geothrix*; *Desulfovibrio*; *Desulfobulbus*; and *Geobacter*. The relative abundances of *Desulfovibrio*, *Desulfobulbus* and *Geothrix* gradually increased during the three periods in system 3. Higher proportions of denitrifying bacteria, including *Nitrospira*, *Thauera* and *Dechloromonas*, were observed at the anode. Further studies of the metabolic pathways of pollutant removal and electron motion are needed.

## Conflicts of interest

There are no conflicts to declare.

## Supplementary Material

RA-009-C8RA10130B-s001
