# Supplementary material for: Electricity production and the analysis of the anode microbial community in a constructed wetland-microbial fuel cell
Source: RSC Adv. 2019 Jul 10;9(37):21460–72. doi: 10.1039/c8ra10130b (PMC9066182; doi:10.1039/c8ra10130b)

Electronic Supplementary Material (ESI) for RSC Advances.
This journal is © The Royal Society of Chemistry 2019

Supplementary Information

Table

Table S1. Composition of synthetic wastewaters during period 1.

Component	complete wastewater(mg/L)	lacking ammonium wastewater(mg/L)	lacking nitrate wastewater(mg/L)
$C_6H_{12}O_6 \cdot H_2O$	128.64±0.99	128.64±0.99	128.64±0.99
KH_2PO_4	8.23±0.79	8.23±0.79	8.23±0.79
$Na_2HPO_4 \cdot 12H_2O$	19.08±1.10	19.08±1.10	19.08±1.10
$MgCl_2 \cdot 6H_2O$	18.92±1.06	18.92±1.06	18.92±1.06
$ZnCl_2$	8.11±0.62	8.11±0.62	8.11±0.62
$CaCl_2$	18.79±0.72	18.79±0.72	18.79±0.72
CH_3COONa	107.25±1.20	107.25±1.20	107.25±1.20
$CuSO_4 \cdot 5H_2O$	10.70±0.57	10.70±0.57	10.70±0.57
$NaNO_3$	29.50±0.99	29.50±0.99	-
NH_4Cl	31.60±0.22	-	31.60±0.22

Table S2. Composition of synthetic wastewaters during period 2.

Component	complete wastewater(mg/L)	lacking ammonium wastewater(mg/L)	lacking nitrate wastewater(mg/L)
$C_6H_{12}O_6 \cdot H_2O$	257.18±0.92	257.18±0.92	257.18±0.92
KH_2PO_4	8.23±0.79	8.23±0.79	8.23±0.79
$Na_2HPO_4 \cdot 12H_2O$	19.08±1.10	19.08±1.10	19.08±1.10
$MgCl_2 \cdot 6H_2O$	18.92±1.06	18.92±1.06	18.92±1.06
$ZnCl_2$	8.11±0.62	8.11±0.62	8.11±0.62
$CaCl_2$	18.79±0.72	18.79±0.72	18.79±0.72
CH_3COONa	214.32±0.80	214.32±0.80	214.32±0.80
$CuSO_4 \cdot 5H_2O$	10.70±0.57	10.70±0.57	10.70±0.57
$NaNO_3$	58.93±0.55	58.93±0.55	-
NH_4Cl	63.20±0.46	-	63.20±0.46

Table S3. Composition of synthetic wastewaters during period 3

Component	complete wastewater(mg/L)	lacking ammonium wastewater(mg/L)	lacking nitrate wastewater(mg/L)
$C_6H_{12}O_6 \cdot H_2O$	514.33±0.15	514.33±0.15	514.33±0.15
KH_2PO_4	8.23±0.79	8.23±0.79	8.23±0.79
$Na_2HPO_4 \cdot 12H_2O$	19.08±1.10	19.08±1.10	19.08±1.10
$MgCl_2 \cdot 6H_2O$	18.92±1.06	18.92±1.06	18.92±1.06
$ZnCl_2$	8.11±0.62	8.11±0.62	8.11±0.62
$CaCl_2$	18.79±0.72	18.79±0.72	18.79±0.72
CH_3COONa	428.60±0.43	428.60±0.43	428.60±0.43
$CuSO_4 \cdot 5H_2O$	10.70±0.57	10.70±0.57	10.70±0.57
$NaNO_3$	117.83±0.47	117.83±0.47	-
NH_4Cl	102.73±0.58	-	102.73±0.58

Table S4. CE of three systems during three periods

	System 1(%)	System 2(%)	System 3(%)
Period 1	0.5-0.62	0.3-0.42	0.52-0.85
Period 2	0.46-0.59	0.38-0.47	0.5-0.47
Period 2	0.2-0.31	0.16-0.22	0.3-0.41

Figure

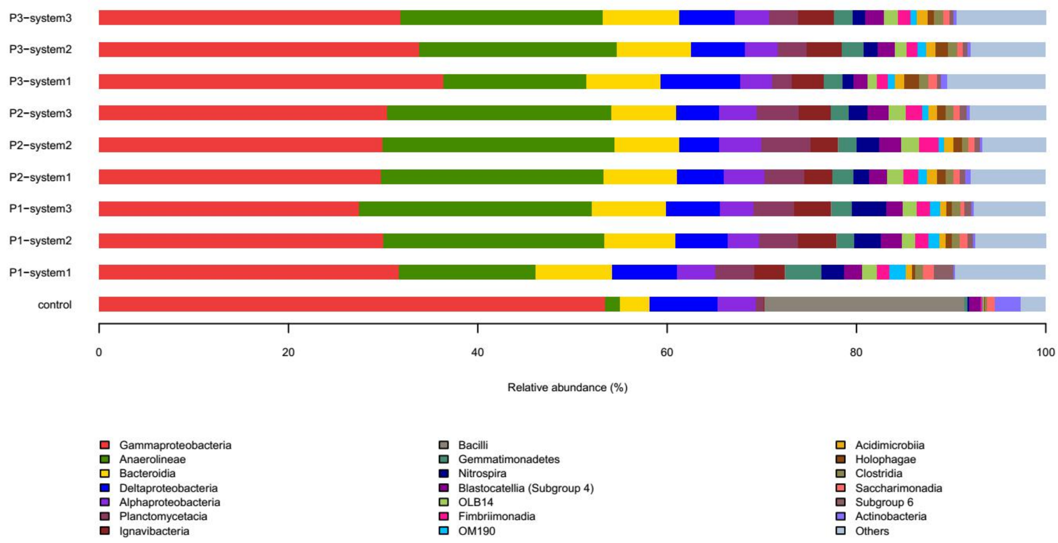

Fig S1. Relative abundance of 16S rDNA sequences of the anode biofilms in CW-MFCs supplemented using different types of synthetic wastewater during three periods at class levels .

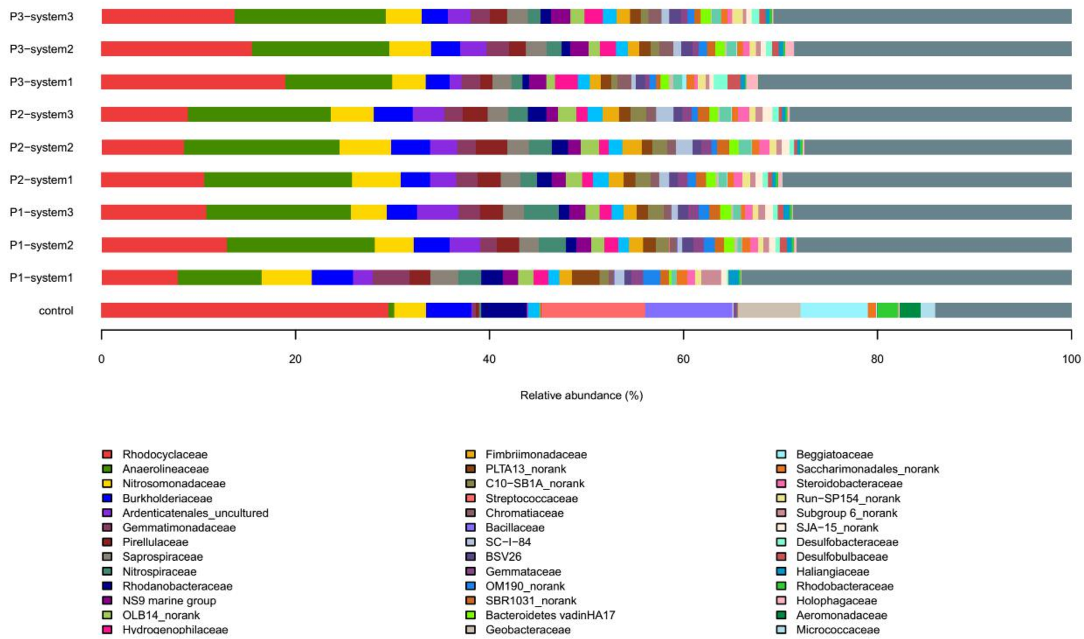

Fig S2. Relative abundance of 16S rDNA sequences of the anode biofilms in CW-MFCs supplemented using different types of synthetic wastewater during three periods at family levels .

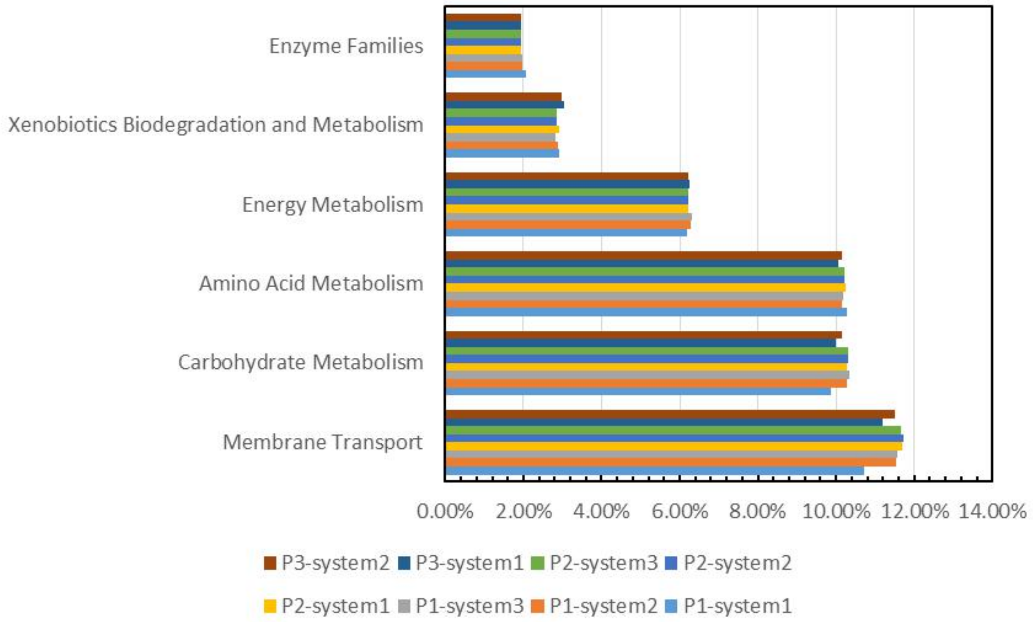

Fig S3. Prediction of the abundance of functional gene contents of the anode biofilms in

CW-MFCs supplemented using different types of synthetic wastewater during three periods.

A

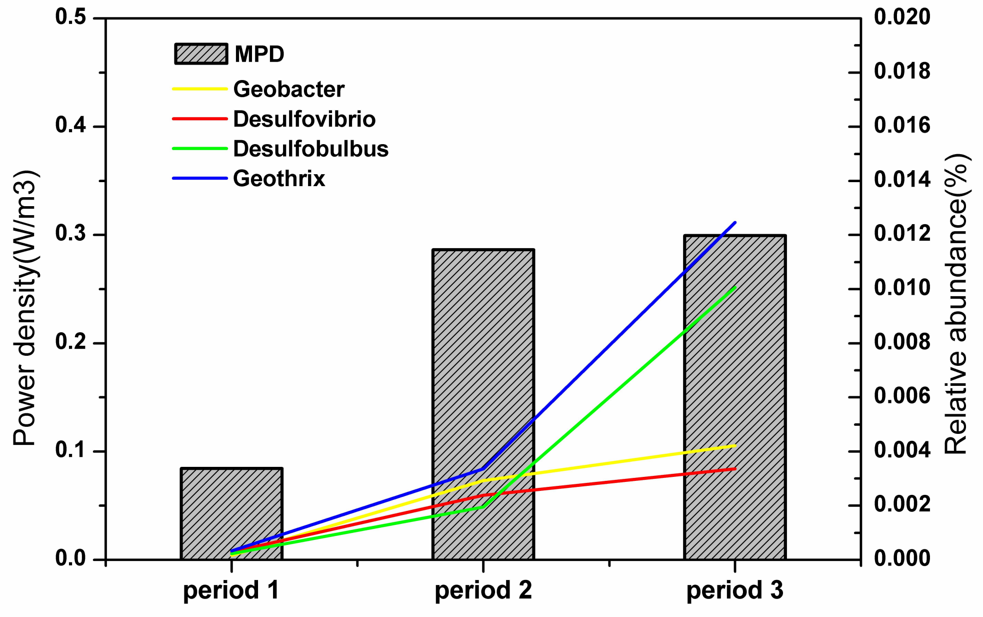

B

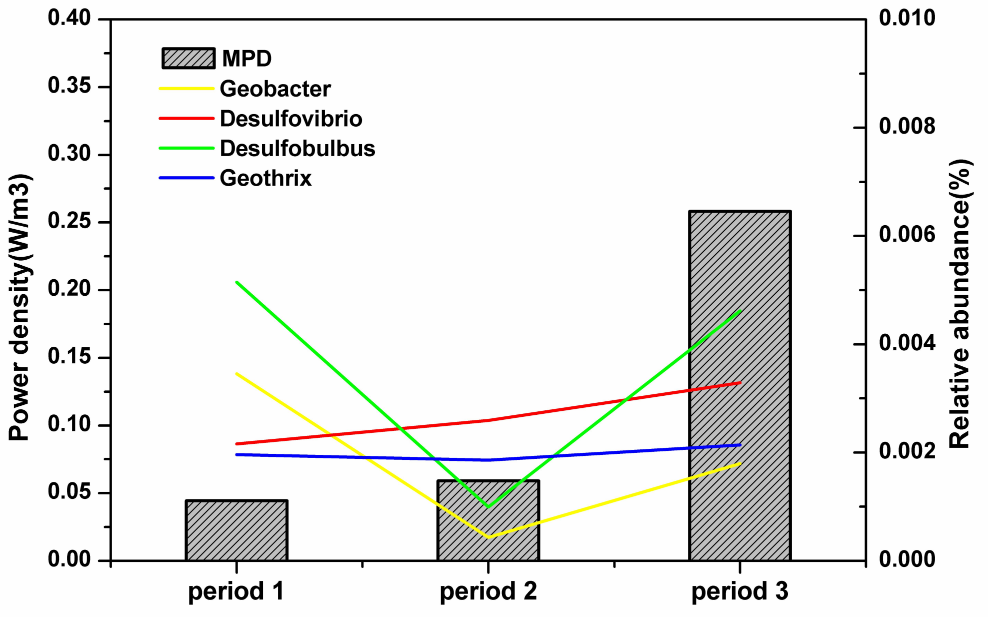

C

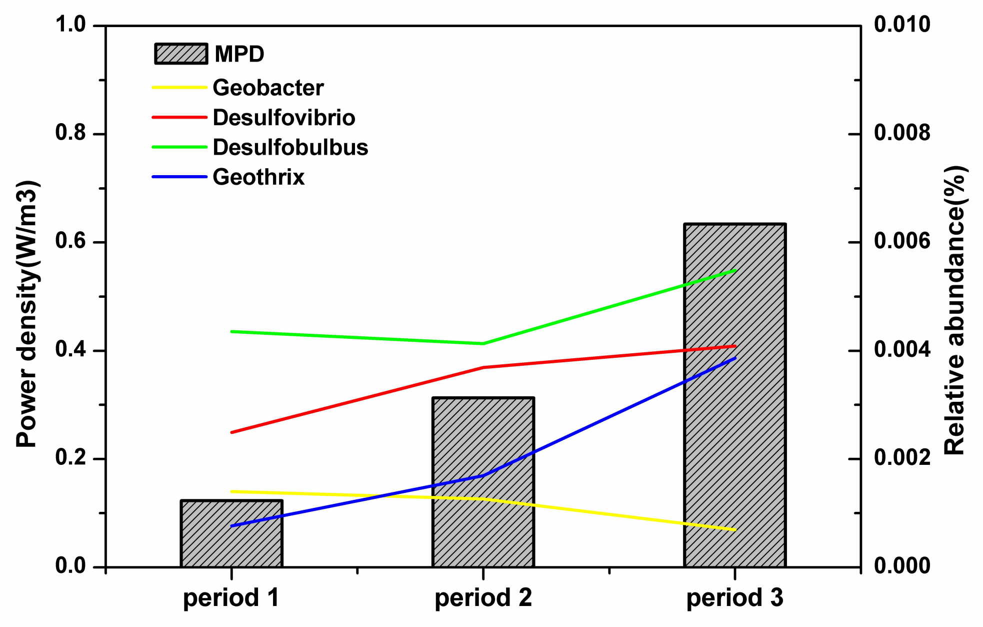

Fig S4 Correlation between maximum power density and the relative abundance of four important EAB. (A) (B) and (C) represent this correlation in system 1, system 2 and system 3 during three periods.

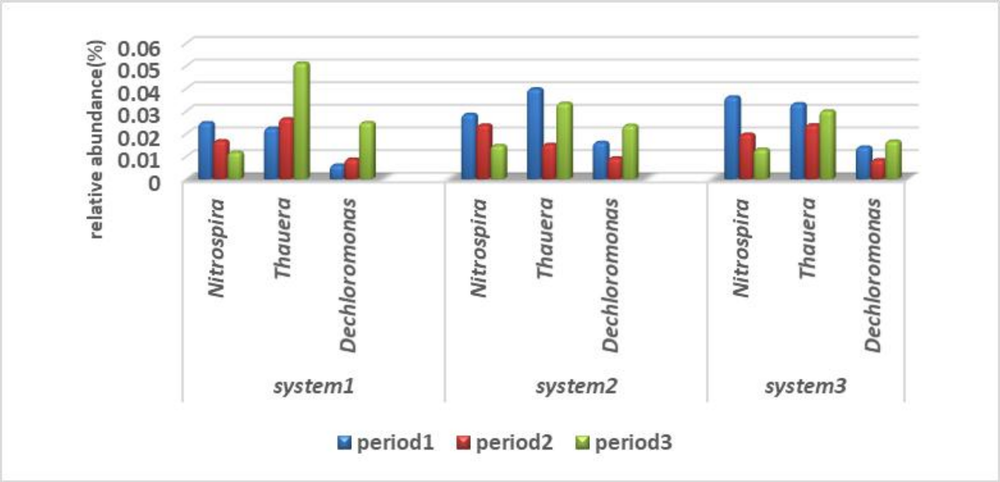

Fig S5 The relative abundance of three important denitrifying bacteria during three systems.

graphical abstract

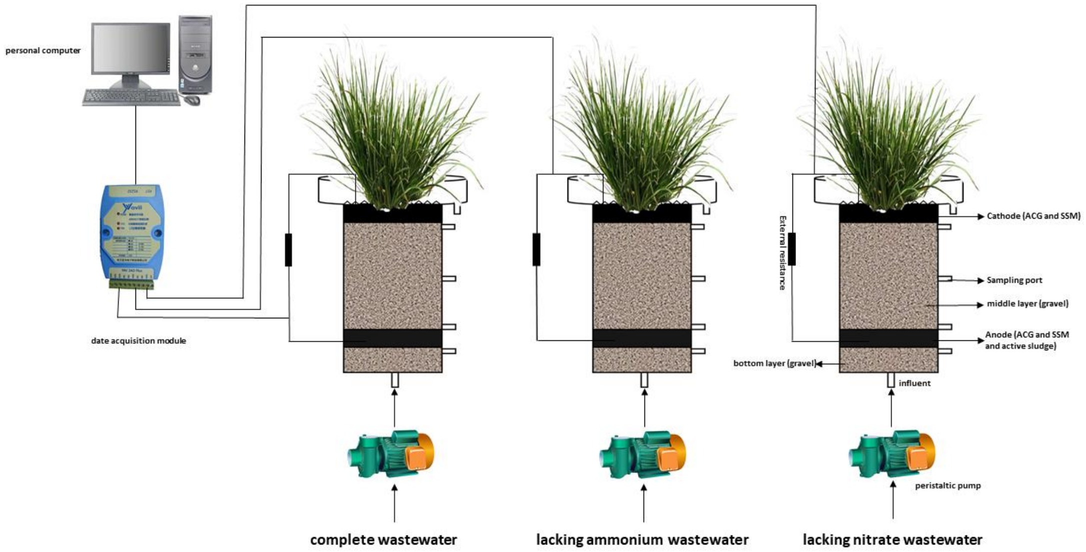

Supplement: RA-009-C8RA10130B-s001 [file RA-009-C8RA10130B-s001.pdf]
